# Supplementary material for: The Effect of Dicer Knockout on RNA Interference Using Various Dicer Substrate Small Interfering RNA (DsiRNA) Structures
Source: Genes (Basel). 2022 Feb 27;13(3):436. doi: 10.3390/genes13030436 (PMC8952432; doi:10.3390/genes13030436)
Supplement: Supplementary file 1 [file genes-13-00436-s001.zip › genes-1534842-supplementary.pdf]

Supplementary Materials:

Figure S1. gDNA PCR from single-clone cells.

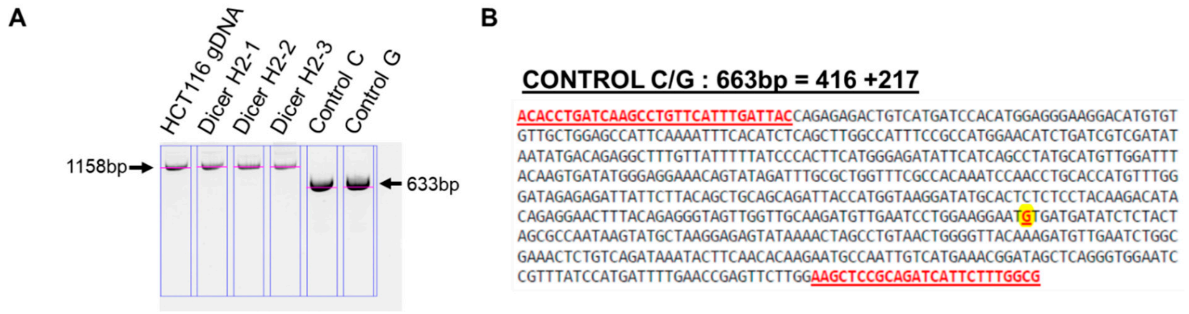

A. PCR using gDNA from single-cell clones of H2-1, H2-2, and H2-3 (1158 bp), control C, and control G (633 bp). **B**. Control DNA sequence. Yellow highlighted G is the point mutation site, which is either G or C. Underlined red letters indicate forward and reverse primer sites for gDNA PCR

Figure S2. The analysis of genomic DNA and amino acid in H2-2 and HCT116.

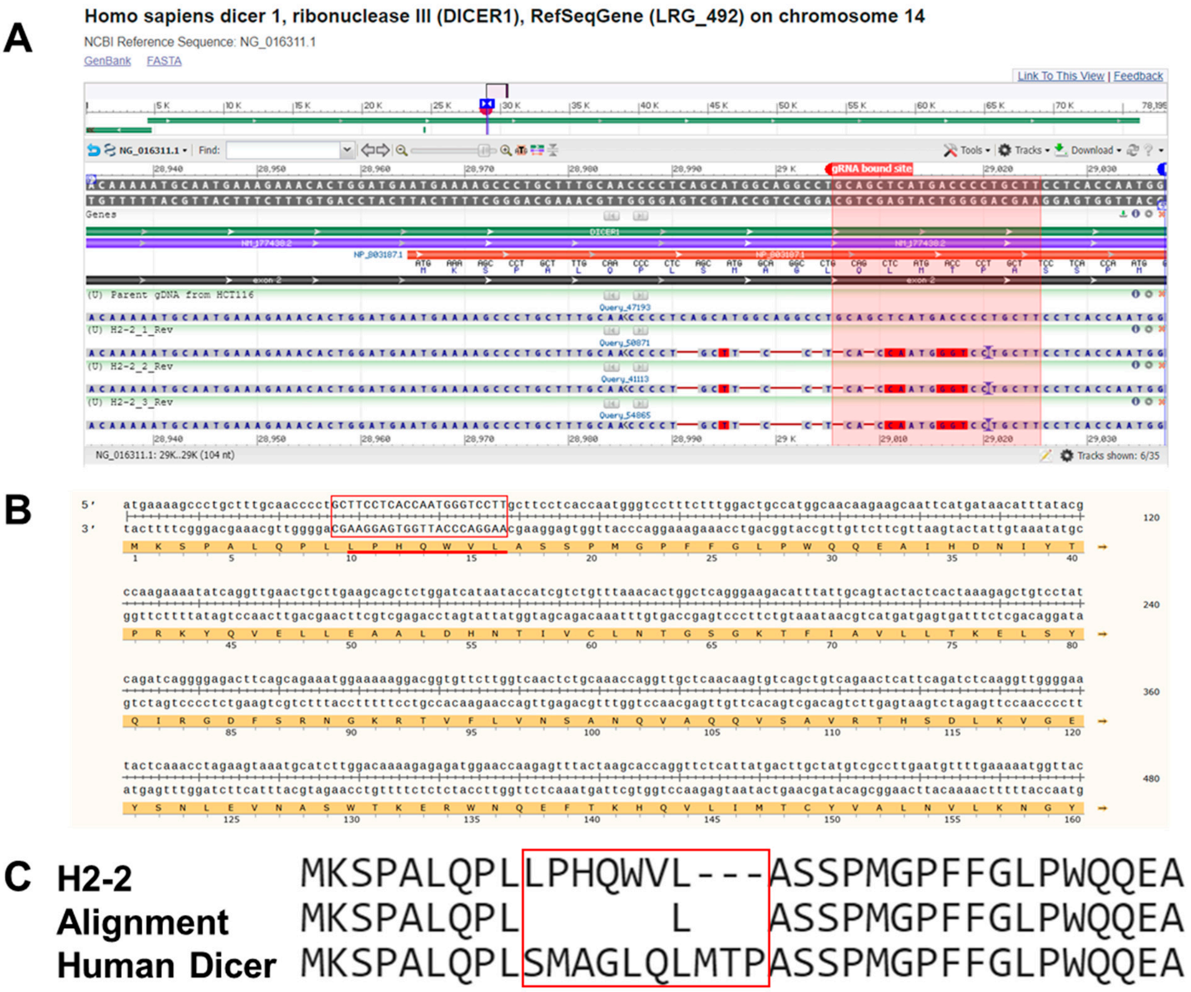

A. Sequencing alignment of Dicer gDNA(XP_01687661 : NCBI reference sequence), HCT116 clone, and H2-2 clone sequences. NCBI blast RID number is HCT116 clone (ZM5P6T54013) and H2-2 clones (ZM5U6DD3013, ZM5Y4U2P013, and ZM62W9CX016). **B**. The H2-2 amino acid sequence. **C**. Sequence alignment comparing the human Dicer amino acid sequence and H2-2 amino acid sequence. Red box denotes the mutation sequence. There are six amino acid mutations and three amino acid deletions.

Figure S3. Sequencing analysis of PCR bands.

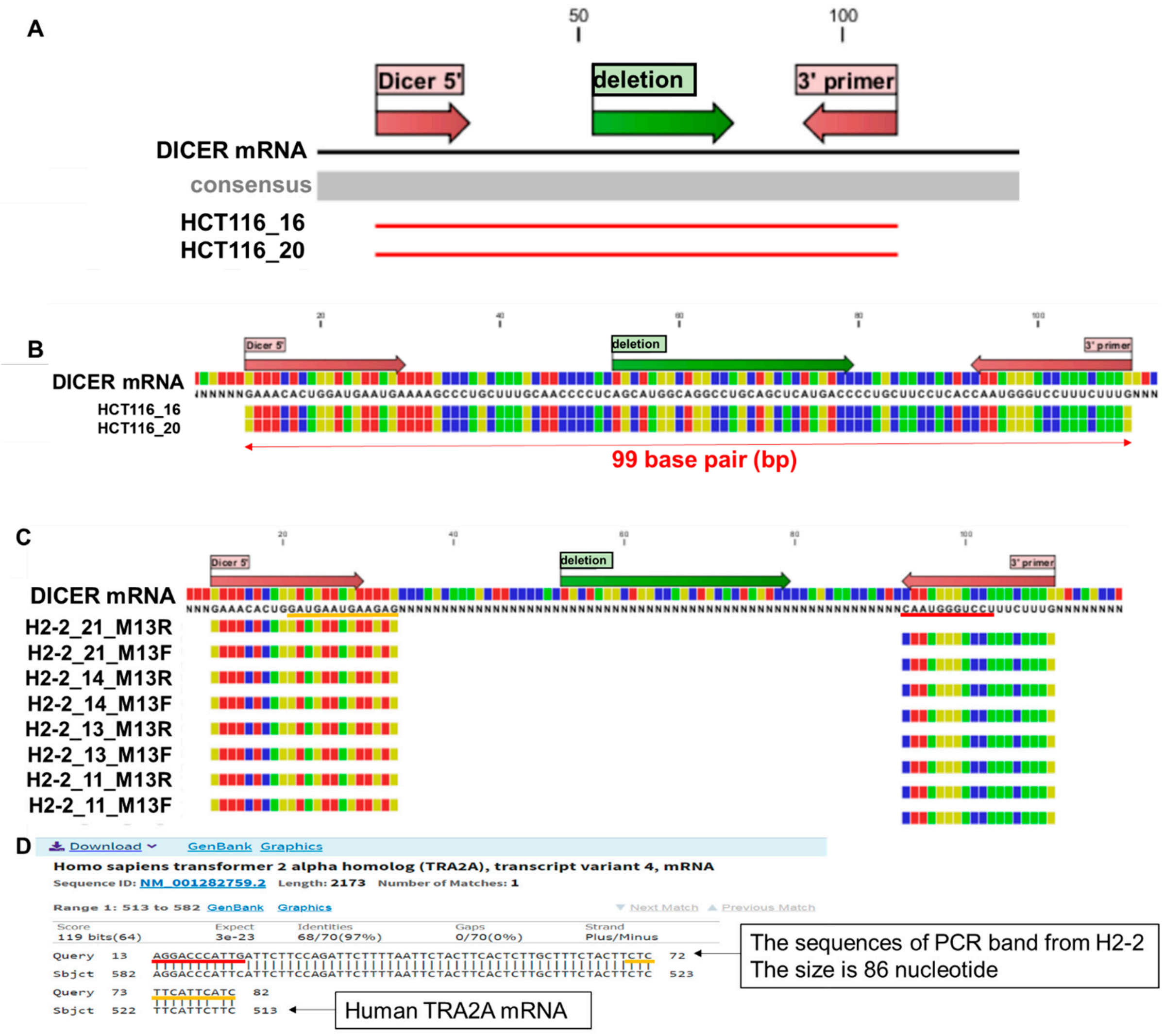

A. Schematic of DNA sequencing results of PCR bands from HCT116 cell colonies. Colonies 16 and 20 perfectly matched the Dicer mRNA (NM_001195573). **B**. The Dicer sequences from HCT116 colonies 16 and 20 had 99 nucleotides. **C**. The PCR bands from H2-2 clones numbered 21, 14, 13, and 11 did not align with Dicer mRNA, represented by N. Only the primer areas showed alignment with the Dicer mRNA sequence (orange and red underline). **D**. NCBI nucleotide alignment shows that the PCR band from H2-2 is a non-specific band from transformer two alpha homolog (TRA2A) mRNA. The TRA2A mRNA sequence aligned with the forward and reverse Dicer primers (red and orange underline). The band size is 86 nucleotides. Guanine: Yellow squares, Adenosine: Red squares, Cytidine: Blue squares, and Uridine: Green squares.

Figure S4. miRNAs expression in parent cells (HCT116) and Dicer knockout cells (H2-2).

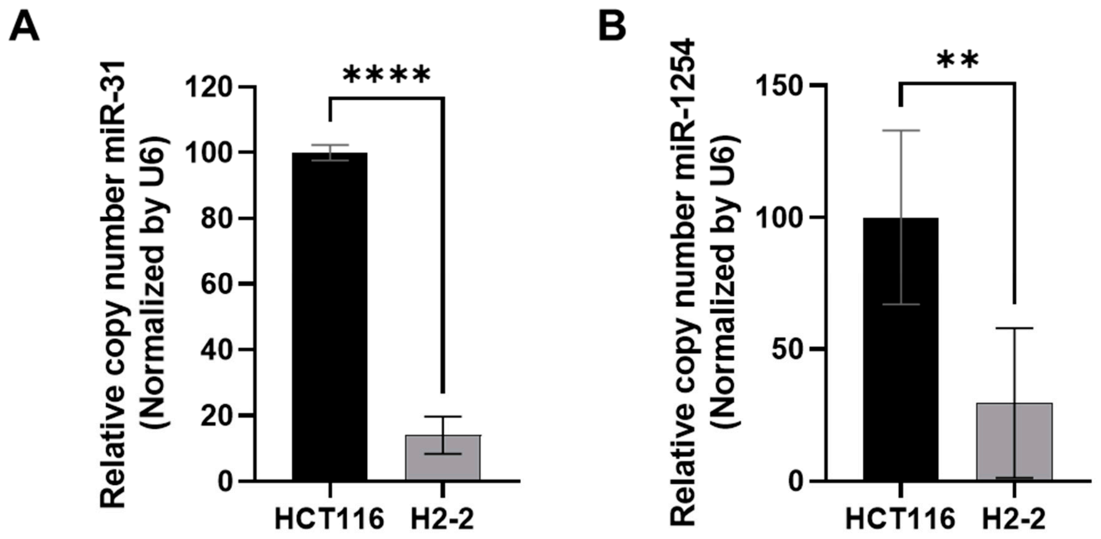

Copy number of miR-31(**A**) and miR-1254(**B**) relative to HCT116 in the normalized expression of U6 measured by TaqMan™ miRNA assay (Applied Biosystems™ catalog number:4427975). Three independent biological replicates were analyzed. Data are represented as mean ± SD. Student's t-test **P<0.001 and ****P<0.0001.

Figure S5. Gene silencing activity in each strand of DsiRNA.

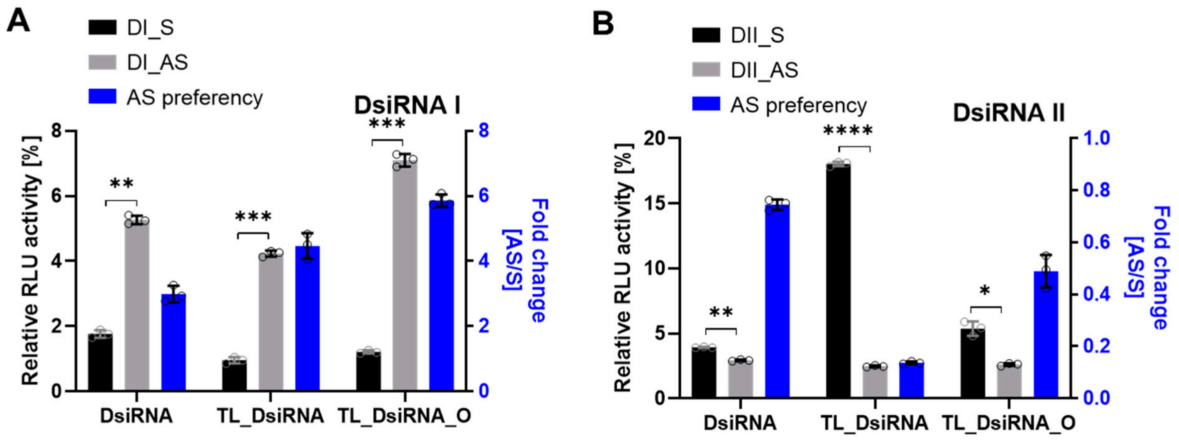

DsiRNA I (**A**) and DsiRNA II (**B**) transfected HCT116 cells were lysed in 1X passive lysis buffer, and dual-luciferase activities were determined. We calculated AS preference (blue bar) as Relative Rlue activity of AS divided by S activity. DsiRNA I showed more than 2-fold AS preference. In contrast, the AS preference of DsiRNA II was below 1; DsiRNA IIs showed preference for the S strand in DsiRNA II, TL_DsiRNA II, and TL¬_DsiRNA II_O. The data shown represent the mean ± S.D. of three independent experiments (Student's t-test *P<0.05, **P<0.01, ***P<0.001, and ****P<0.0001).

Table S1. Peptide identification results of Dicer with altered protein abundance in H2-2 with deletion of Dicer and parental cells in HCT116.

Sample	Unique Peptides	Protein quantitated	Total #PSMs	Peptide lengths	Confidence
HCT116	3	YES	4	19	High
				19	
				12	
H2-2	0	NO	0	-	-
